# Region-specific effects on brain metabolites of hypoxia and hyperoxia overlaid on cerebral ischemia in young and old rats: a quantitative proton magnetic resonance spectroscopy study

**DOI:** 10.1186/1423-0127-17-14

**Published:** 2010-02-23

**Authors:** Maria A Macri, Nicola D'Alessandro, Camillo Di Giulio, Patrizia Di Iorio, Silvano Di Luzio, Patricia Giuliani, Ennio Esposito, Mieczyslaw Pokorski

**Affiliations:** 1 Department of Experimental Medicine and Pathology, "La Sapienza" University, Rome and S Lucia Foundation, Rome, Italy; 2 Department of Sciences, "G D'Annunzio" University of Chieti-Pescara, Italy; 3 Department of Basic and Applied Medical Sciences, "G D'Annunzio" University of Chieti-Pescara, Italy; 4 Department of Human Movement Sciences, "G D'Annunzio" University of Chieti-Pescara, Italy; 5 Deparment of Clinical Sciences and Bioimaging, "G D'Annunzio" University of Chieti-Pescara, Italy; 6 Istituto di Ricerche Farmacologiche "Mario Negri", Consorzio "Mario Negri" Sud, Santa Maria Imbaro, Chieti, Italy; 7 Department of Respiratory Research, Medical Research Center, Polish Academy of Sciences, Warsaw, Poland

## Abstract

**Background:**

Both hypoxia and hyperoxia, deregulating the oxidative balance, may play a role in the pathology of neurodegenerative disorders underlain by cerebral ischemia. In the present study, quantitative proton magnetic resonance spectroscopy was used to evaluate regional metabolic alterations, following a 24-hour hypoxic or hyperoxic exposure on the background of ischemic brain insult, in two contrasting age-groups of rats: young - 3 months old and aged - 24 months old.

**Methods:**

Cerebral ischemia was induced by ligation of the right common carotid artery. Concentrations of eight metabolites (alanine, choline-containing compounds, total creatine, γ-aminobutyric acid, glutamate, lactate, myo-inositol and N-acetylaspartate) were quantified from extracts in three different brain regions (fronto-parietal and occipital cortices and the hippocampus) from both hemispheres.

**Results:**

In the control normoxic condition, there were significant increases in lactate and myo-inositol concentrations in the hippocampus of the aged rats, compared with the respective values in the young ones. In the ischemia-hypoxia condition, the most prevalent changes in the brain metabolites were found in the hippocampal regions of both young and aged rats; but the effects were more evident in the aged animals. The ischemia-hyperoxia procedure caused less dedicated changes in the brain metabolites, which may reflect more limited tissue damage.

**Conclusions:**

We conclude that the hippocampus turns out to be particularly susceptible to hypoxia overlaid on cerebral ischemia and that old age further increases this susceptibility.

## Background

It is well established that mitochondrial dysfunction and oxidative damage are essential in the slowly progressive neuronal death that is characteristic of aging and neurodegenerative disorders, including Alzheimer and Parkinson's diseases [1-3]. The brain, which consumes large amounts of oxygen, is particularly vulnerable to oxidative stress. Antioxidant defense systems can be upregulated in response to increased reactive oxygen species (ROS) [1]. Although these systems may confer protection against ROS, they are not fully effective in preventing oxidative damage. Moreover, efficiency of gene expression may decline or become defective with progressive age, as oxidative damage to the genome increases, which diminishes the enzymatic antioxidant efficiency [4,5]. Oxidative stress is considered the prevalent mechanism by which impaired cerebral blood flow, hypoxia, and hyperoxia all cause neuronal damage at the mitochondrial level due to increased ROS production that overwhelms the antioxidant capacity [6-8]. In addition, evidence accumulates that reduced cerebral blood flow plays a role in the pathogenesis of Alzheimer's disease [9] and contributes to cognitive decline which is usually present during aging [10].

On the basis of the above outlined considerations, the present study was designed to test whether the ischemia-induced metabolic impairment would be affected by varying oxygen supply due to hypoxia or hyperoxia in the rat brain. We addressed this issue by measuring the concentrations of selected metabolites, using proton magnetic resonance spectroscopy (^1^H-MRS), in two contrastingly different age-groups of animals: young - 3 months old and aged - 24 months old rats. Furthermore, we sought to determine whether age, in itself, affects the level of brain metabolites. In general, the study demonstrates that hypoxia, overlaid on cerebral ischemia, was a dedicatedly stronger detriment to the brain metabolite content than was hyperoxia in both young and old animals. The hippocampus appeared particularly susceptible to hypoxia-ischemia perturbation and old age further increased this susceptibility.

## Methods

### Animals and ischemic procedure

All procedures were performed in accordance with the guidelines of EC Directive 86/609/EEC for animal experiments and the study protocol was approved by a local Ethics Committee.

A total of 60 adult female Wistar rats were used for the main experiments. Additional 26 rats of either sex were used for a preliminary phase of the study in which gender differences in the survival rate during prolonged hypoxic and hyperoxic exposures after antecedent ischemic brain insult and the control brain content of metabolites without ischemia were assessed, as outlined below. The 60 animals were divided into two contrasting age-groups: young - 3 months old (the mean ± SD weight of 230 ± 20 g) and aged - 24 months old (280 ± 30 g), each consisting of 30 rats. Either age-group was further subdivided into three subgroups of 10 rats each. Two of these subgroups were anesthetized with Nembutal (30 mg·kg^-1^, i.p.), after overnight fast, and were subjected to the ischemic procedure consisting of surgical ligation of the right carotid artery. One each of these subgroups was then hypoxia or hyperoxia-treated. The animals of the remaining third subgroup in either age category were used for basal, control measurements of brain metabolites and, therefore, were intact and untreated.

### Gender differences - preliminary experiments

The choice of female rats for the main part of the study was preceded by preliminary experiments in which the possible gender-related differences in endurance to prolonged hypoxia and hyperoxia applied against the background of cerebral ischemia induced by unilateral common carotid artery occlusion, as outlined below, were investigated. In this phase of the study 16 additional rats were used; 8 of either sex. Six out of the 8 male rats died during ischemia-hypoxia, whereas no mortality was noted among female rats. This result prompted us to continue the study in female rats only, even though the hyperoxia-ischemia procedure did not cause any mortality in either male or female rats.

Moreover, in additional 10 female rats we investigated the effects of hypoxia alone (12% inspired O_2_), without the antecedent cerebral ischemia, on the content of the brain metabolites measured (see the methodological details below). We found that hypoxia, in itself, did not significantly perturb the content of the metabolites. Thus, the levels of metabolites found in the normoxic rats were taken as basal control for those animals that were subjected to ischemia-hypoxia and ischemia-hyperoxia procedures.

### Induction of hypoxia and hyperoxia against the background of ischemia

After the ischemic injury, the rats of the young and aged subgroups, breathed unassisted in Plexiglas chambers for 24 h in hypoxia (12% inspired O_2 _in N_2_) or hyperoxia (100% O_2_) at 23°C. The chambers were recirculated with a pump; CO_2 _was continuously monitored by a capnograph and its excess was removed from the chamber air with Bara Lyme. Boric acid was mixed with the litter to minimize the emission of urinary ammonia. The remaining, control subgroups of rats, in either age-group, were subjected to the same experimental procedures, except the ischemic injury, and breathed normal air instead of hypoxic or hyperoxic gas mixtures. At the end of the exposure period, all rats were decapitated and, in two minutes, three different parts of brain tissue, fronto-parietal and occipital cortices and hippocampus from both hemispheres were removed, weighed, frozen in liquid nitrogen, and stored at -80°C.

### Sample preparation

Perchloric acid (PCA) tissue extracts from the brain areas outlined above were made as described elsewhere [11] and analyzed separately. Briefly, each brain area was homogenized at 5 ml/g in an ice-cold 0.1 M PCA-D_2_O solution. The homogenates were centrifuged at 15000 × *g *for 15 min at 0°C. The supernatant was kept, and the pellet was resuspended in the same original volume of buffer, homogenized, and centrifuged once more, as outlined above. The two supernatants were pooled and 600 μl of the final solution were used for ^1^H-MRS study. Extracts were made from both hemispheres. Thus, a set of six samples was prepared from each animal for the subsequent spectroscopic measurement.

### Brain metabolites and data acquisition

We focused on the brain metabolites, detectable in the proton spectra, which are identifiable at clinical magnetic field strengths and undergo changes in response to ischemia-hypoxia, as found in our previous study (12), and could likely respond also to ischemia-hyperoxia treatment. The following metabolites were quantified: alanine (Ala), choline-containing compounds (Cho), total creatine (Cre), γ-aminobutyric acid (GABA), glutamate (Glu), glutamine (Gln), lactate (Lac), myo-inositol (mI) and N-acetylaspartate (NAA). Since Cre, usually considered as an internal concentration reference in various pathological states, could not be used for this purpose due to its potential variability in the experimental model used, a solution of 3-(trimethylsilyl)-2,2',3,3'-tetradeuteropropionic acid (TMSP-d_4_) was used as an external standard for the quantitative measurements in the present investigation.

Proton magnetic resonance spectra were acquired with an AVANCE NMR spectrometer (Bruker BioSpin, Milan, Italy), using a pulse-acquired sequence at 300 MHz at 7.05 T and temperature of 300 K. Typical parameters used for data acquisition consisted of a sweep width of 4 kHz, 16 K sample points, TR = 10 s, and 120 scans. Water suppression was achieved by applying a Bruker-made pulse sequence. Extracts (600 μl) were inserted in a 5 mm MRS tube. A coassial insert containing a solution of 30 mM TMSP-d_4 _in D_2_O was used as an external standard in each spectroscopic investigation. The quantification of metabolites in brain extracts was preceded by a preliminary work performed on a set of individual metabolite solutions and a mixture model solution, containing the chemical species of interest (all chemicals purchased from Sigma Chemical Company, St. Louis, MO). The model solution contained known amounts of NAA (100 mM), Ala (10 mM), Cre (100 mM), GABA (100 mM), Glu (100 mM), Lac (25 mM), mI (100 mM), and phosphoric acid (50 mM) in 0.1 M D_2_O. Pure compound solutions were prepared in the same way. Special care was devoted to pH of solutions, which was adjusted to 1.5, since it is critical for a quantitative MRS evaluation of brain extracts [12,13]. Attention was paid to keep the post-mortem changes of brain tissue to a minimum, to avoid increases in tissue lactate and GABA levels [14].

### Data processing

Proton assignments were made by comparing resonances of individual D_2_O solutions of the metabolites under investigation at the same pH value of the extracts [15]. All chemical shifts were referenced to the TMSP-d_4 _signal at 0.0 ppm. The peak areas were determined by the integration of the identified resonances and were normalized to the TMSP-d_4 _signal area. Solutions of glycine (Gly), ranging from 3.0 to 60 μmol, were prepared and MRS-investigated to establish a calibration curve, against which the concentrations of the measured metabolites were evaluated. The volume of each Gly solution was adjusted to a total volume of 600 μl. The integral values of Gly were normalized to the 30 mM TMSP-d_4 _solution contained in a coassial capillary inserted into the MRS tube. The ratio of peak area of each metabolite to signal area of TMSP-d_4 _was fitted to the linear concentration curve of Gly. Finally, the absolute quantity of a metabolite (in mmol/kg wet weight) was corrected by normalizing the number of its protons to the number of Gly protons (2H).

### Statistical analysis

Two-way ANOVA was used to test the effects of age, treatment (ischemia-hypoxia and ischemia-hyperoxia) or age × treatment, for each metabolite in the three brain regions studied and for both hemispheres. The differences contributed by the age factor following the two different treatment conditions were further analyzed by a parametric (unpaired *t*-test) or nonparametric (Mann-Whitney U test) method. The Bonferroni correction was applied to account for multiple comparisons. P < 0.05 was considered significant in all statistical evaluations.

## Results

### Basal brain levels of metabolites in young and aged rats

In the control untreated age-groups, i.e., young and aged normoxic rats, as opposed to the experimentally treated groups outlined in the paragraphs below, statistical analysis revealed no significant difference between the two brain hemispheres; therefore, the concentration of each metabolite was averaged from the pooled data representing the symmetric brain areas in each rat. There were neither intragroup nor intergroup statistical differences in the basal concentrations of the corresponding metabolites in the fronto-parietal and occipital cortices. However, Ala and Lac were appreciably higher in the hippocampus of the young rats compared with the respective values in the cortices (P < 0.05). In addition, the Lac and mI levels were higher in the hippocampus of the aged rats compared with the respective values in the young rats (P < 0.05) (see control columns in Tables 1, 2, 3, 4, 5 and 6).

**Table 1:** Effects of ischemia-hypoxia and ischemia-hyperoxia in the fronto-parietal cortex of young rats

		Ischemia-hypoxia		Ischemia-hyperoxia	
		
Metabolite	Control	Right	Left	Right	Left
Alanine	0.66 ± 0.05	0.60 ± 0.03	0.66 ± 0.09	0.39 ± 0.07*	0.60 ± 0.08
Choline	3.74 ± 0.22	3.47 ± 0.13	3.88 ± 0.15	3.73 ± 0.26	3.58 ± 0.16
Creatine	10.99 ± 0.50	11.90 ± 0.10	12.78 ± 0.75	11.44 ± 0.65	11.43 ± 0.44
GABA	2.16 ± 0.11	1.40 ± 0.08**	1.67 ± 0.34	1.53 ± 0.28*	1.88 ± 0.28
Glutamate	14.44 ± 0.45	13.88 ± 0.45*	15.96 ± 0.93	13.78 ± 0.80	13.98 ± 0.70
Lactate	11.88 ± 0.28	15.18 ± 0.29**	17.01 ± 1.00**	13.55 ± 1.10*	13.93 ± 0.23**
Myo-inositol	4.98 ± 0.62	6.47 ± 0.80	7.36 ± 0.40*	6.74 ± 0.26*	5.63 ± 0.24
N-acetylaspartate	10.29 ± 0.16	10.33 ± 0.40	12.08 ± 0.50**	10.41 ± 0.65	10.64 ± 0.60

Concentrations of metabolites (mmol/kg w/w) in young rats, under normoxic condition (control), ischemia-hypoxia, and ischemia-hyperoxia. Data are means ± SE (n = 10 rats/group). Values, determined by proton MRS as outlined in the Methods, are given for the right (ipsilateral to cerebral ischemic injury) and left (contralateral) hemisphere. *P < 0.05 and **P < 0.01 compared with controls.

**Table 2:** Effects of ischemia-hypoxia and ischemia-hyperoxia in the fronto-parietal cortex of aged rats

		Ischemia-hypoxia		Ischemia-hyperoxia	
		
Metabolite	Control	Right	Left	Right	Left
Alanine	0.63 ± 0.07	0.59 ± 0.04	0.58 ± 0.06	0.93 ± 0.34	0.66 ± 0.03
Choline	3.43 ± 0.09	3.74 ± 0.08	3.87 ± 0.23	3.58 ± 0.15	4.01 ± 0.09
Creatine	11.63 ± 0.24	11.67 ± 0.35	12.49 ± 0.39	10.80 ± 0.35	11.88 ± 0.21
GABA	1.79 ± 0.18	1.28 ± 0.08*	1.54 ± 0.31	1.13 ± 0.35*	2.18 ± 0.25
Glutamate	15.03 ± 0.26	15.09 ± 0.45	15.92 ± 0.80	12.52 ± 0.80**	15.28 ± 0.90
Lactate	12.59 ± 0.30	16.43 ± 0.40***	16.45 ± 0.83***	15.91 ± 1.00**	15.66 ± 0.76**
Myo-inositol	5.63 ± 0.46	6.64 ± 0.25	7.02 ± 0.50*	6.11 ± 0.50	6.67 ± 0.33*
N-acetylaspartate	10.69 ± 0.21	11.53 ± 0.37	11.66 ± 0.37	9.65 ± 0.32	11.09 ± 0.36

Concentrations of metabolites (mmol/kg w/w) in aged rats, under normoxic condition (control), ischemia-hypoxia, and ischemia-hyperoxia. Data are means ± SE (n = 10 rats/group). Values, determined by proton MRS as outlined in the Methods, are given for the right (ipsilateral to cerebral ischemic injury) and left (contralateral) hemisphere. *P < 0.05, **P < 0.01, and ***P < 0.001 compared with controls.

**Table 3:** Effects of ischemia-hypoxia and ischemia-hyperoxia in the occipital cortex of young rats

		Ischemia-hypoxia		Ischemia-hyperoxia	
		
Metabolite	Control	Right	Left	Right	Left
Alanine	0.64 ± 0.04	0.76 ± 0.08	0.73 ± 0.19	0.73 ± 0.02	0.50 ± 0.04
Choline	3.75 ± 0.05	3.59 ± 0.06	3.88 ± 0.23	4.34 ± 0.25	4.32 ± 0.29
Creatine	10.64 ± 0.10	11.81 ± 0.25**	12.08 ± 0.61	12.97 ± 0.60*	13.21 ± 1.00*
GABA	1.72 ± 0.21	1.76 ± 0.20	2.06 ± 0.54	2.52 ± 0.50*	2.02 ± 0.28
Glutamate	14.55 ± 0.18	14.91 ± 0.29	15.58 ± 1.50	16.17 ± 0.80*	16.02 ± 0.60*
Lactate	12.10 ± 0.51	16.56 ± 0.49***	17.87 ± 0.33***	16.03 ± 1.20**	15.73 ± 0.85**
Myo-inositol	5.67 ± 0.08	8.56 ± 0.40*	8.18 ± 1.14*	8.42 ± 0.90**	8.22 ± 0.80**
N-acetylaspartate	10.15 ± 0.21	11.01 ± 0.62	11.47 ± 0.85	11.85 ± 0.41	11.53 ± 0.44

Concentrations of metabolites (mmol/kg w/w) in young rats, under normoxic condition (control), ischemia-hypoxia, and ischemia-hyperoxia. Data are means ± SE (n = 10 rats/group). Values, determined by proton MRS as outlined in the Methods, are given for the right (ipsilateral to cerebral ischemic injury) and left (contralateral) hemisphere. *P < 0.05, **P < 0.01, and ***P < 0.001 compared with controls.

**Table 4:** Effects of ischemia-hypoxia and ischemia-hyperoxia in the occipital cortex of aged rats

		Ischemia-hypoxia		Ischemia-hyperoxia	
		
Metabolite	Control	Right	Left	Right	Left
Alanine	0.61 ± 0.06	0.72 ± 0.03	0.85 ± 0.21	0.88 ± 0.07*	0.57 ± 0.12
Choline	3.50 ± 0.14	3.61 ± 0.07	3.74 ± 0.23	3.94 ± 0.36	3.72 ± 0.07
Creatine	10.53 ± 0.37	10.86 ± 0.24	10.76 ± 0.31	11.31 ± 0.90	11.32 ± 0.46
GABA	1.86 ± 0.24	2.02 ± 0.13	2.11 ± 0.59	3.16 ± 0.60*	2.25 ± 0.33
Glutamate	13.89 ± 0.51	13.73 ± 0.21	13.93 ± 0.46	13.71 ± 1.30	14.06 ± 0.26
Lactate	12.82 ± 0.49	16.12 ± 0.44***	16.37 ± 0.53***	15.53 ± 0.48**	15.69 ± 0.60**
Myo-inositol	6.55 ± 0.18	7.06 ± 0.35	7.76 ± 0.85*	7.63 ± 0.75	7.12 ± 0.33
N-acetylaspartate	9.98 ± 0.36	10.48 ± 0.30	10.87 ± 0.34	10.36 ± 0.60	10.14 ± 0.38

Concentrations of metabolites (mmol/kg w/w) in aged rats, under normoxic condition (control), ischemia-hypoxia, and ischemia-hyperoxia. Data are means ± SE (n = 10 rats/group). Values, determined by proton MRS as outlined in the Methods, are given for the right (ipsilateral to cerebral ischemic injury) and left (contralateral) hemisphere. *P < 0.05, **P < 0.01, and ***P < 0.001 compared with controls.

**Table 5:** Effects of ischemia-hypoxia and ischemia-hyperoxia in the hippocampus of young rats

		Ischemia-hypoxia		Ischemia-hyperoxia	
		
Metabolite	Control	Right	Left	Right	Left
Alanine	0.89 ± 0.08	0.43 ± 0.02*	0.37 ± 0.05*	0.70 ± 0.20	0.85 ± 0.03
Choline	4.11 ± 0.12	2.20 ± 0.45**	2.48 ± 0.11**	3.48 ± 0.45	3.77 ± 0.17
Creatine	11.96 ± 0.20	8.95 ± 0.48*	8.28 ± 0.62**	10.50 ± 1.50	11.08 ± 0.23
GABA	2.12 ± 0.12	0.73 ± 0.16**	0.88 ± 0.24**	2.07 ± 0.35	1.82 ± 0.09
Glutamate	14.93 ± 0.71	9.27 ± 1.50*	8.77 ± 0.81**	12.36 ± 1.50	11.33 ± 0.70**
Lactate	14.63 ± 0.22	12.45 ± 1.50*	12.23 ± 0.80*	16.57 ± 0.45**	15.44 ± 0.15*
Myo-inositol	7.47 ± 0.35	5.26 ± 1.10	5.92 ± 0.20*	7.90 ± 0.80	8.22 ± 0.38
N-acetylaspartate	9.70 ± 0.12	6.88 ± 1.40*	6.81 ± 0.24*	8.67 ± 1.20	8.79 ± 0.32

Concentrations of metabolites (mmol/kg w/w) in young rats, under normoxic condition (control), ischemia-hypoxia, and ischemia-hyperoxia. Data are means ± SE (n = 10 rats/group). Values, determined by proton MRS as outlined in the Methods, are given for the right (ipsilateral to cerebral ischemic injury) and left (contralateral) hemisphere. *P < 0.05 and **P < 0.01 compared with controls.

**Table 6:** Effects of ischemia-hypoxia and ischemia-hyperoxia in the hippocampus of aged rats

		Ischemia-hypoxia		Ischemia-hyperoxia	
		
Metabolite	Control	Right	Left	Right	Left
Alanine	0.91 ± 0.12	0.37 ± 0.05*	0.38 ± 0.07*	1.28 ± 0.36	1.01 ± 0.23
Choline	4.27 ± 0.08	2.56 ± 0.40***	2.69 ± 0.18***	4.03 ± 0.28	4.03 ± 0.26
Creatine	12.69 ± 0.32	7.52 ± 0.90*	7.80 ± 0.75**	12.31 ± 0.41	11.68 ± 0.58
GABA	2.65 ± 0.24	1.38 ± 0.39**	1.66 ± 0.23**	3.22 ± 0.48	2.69 ± 0.31
Glutamate	15.53 ± 0.44	6.97 ± 0.96***	6.88 ± 0.48***	12.67 ± 1.40*	13.06 ± 1.00**
Lactate	16.24 ± 0.34	12.69 ± 1.42***	11.38 ± 0.55***	17.05 ± 0.26*	17.03 ± 1.00*
Myo-inositol	8.26 ± 0.45	5.27 ± 1.15*	5.87 ± 0.66**	8.75 ± 0.50	9.01 ± 0.56
N-acetylaspartate	10.51 ± 0.18	6.35 ± 0.45***	6.00 ± 0.42***	9.41 ± 0.37	9.13 ± 0.46

Concentrations of metabolites (mmol/kg w/w) in aged rats, under normoxic condition (control), ischemia-hypoxia, and ischemia-hyperoxia. Data are means ± SE (n = 10 rats/group). Values, determined by proton MRS as outlined in the Methods, are given for the right (ipsilateral to cerebral ischemic injury) and left (contralateral) hemisphere. *P < 0.05, **P < 0.01, and ***P < 0.001 compared with controls.

### Effects of ischemia-hypoxia and ischemia-hyperoxia in the fronto-parietal cortex

Ischemia-hypoxia significantly increased the amount of Lac in the fronto-parietal cortex of both hemispheres in both young and aged rats (P < 0.001), compared with the respective control groups. In addition, the procedure elicited a significant increase in mI on the left side (P < 0.05) and a decease in GABA on the right side, contralateral and ipsilateral to the ischemic injury, respectively, in both young (P < 0.001) and aged rats (P < 0.05) (Tables 1, 2). There were no appreciable differences in changes of other metabolites due to ischemia-hypoxia between the young and old rats.

Ischemia-hyperoxia also increased the amount of Lac in the fronto-parietal cortex in both young and aged rats, with respect to their age-matched control values, in both right and left hemispheres. In both age-groups, these increases, albeit significant, were somewhat smaller than those found in the corresponding brain areas during ischemia-hypoxia, but the decline in Lac increase in ischemia-hyperoxia was less pronounced in the aged rats (Tables 1, 2). In the young rats, the levels of mI tended to be enhanced on both sides, although this effect, along with reductions in Ala and GABA, was significant only on the right side, ipsilateral to the occlusion (P < 0.05) (Table 1). In contrast, in the old rats the increase in mI during ischemia-hyperoxia reached significance in the contralateral to occlusion fronto-parietal cortex, which was accompanied by decreases in Glu and GABA on the ipsilateral side (Table 2).

### Effects of ischemia-hypoxia and ischemia-hyperoxia in the occipital cortex

Both ischemia-hypoxia and ischemia-hyperoxia induced a significant increase in Lac levels in the occipital cortex in both brain hemispheres of the young and aged rats, compared with the baseline levels (Tables 3, 4). In the young rats, both gas conditions also increased the level of mI (P < 0.05). In these rats ischemia-hyperoxia, but not ischemia-hypoxia, increased the level of Cre and Glu. All these increases were of similar magnitude in both hemispheres. The hyperoxic increase in mI was absent in the aged rats. Ischemia-hyperoxia also increased the content of GABA in both age-groups and that of Ala in the aged group only on the side ipsilateral to occlusion (P < 0.05). The other metabolites remained unchanged in both age-groups (Tables 3, 4).

### Effects of ischemia-hypoxia and ischemia-hyperoxia in the hippocampus

Ischemia-hypoxia had a marked reducing effect on all the metabolites under investigation in the hippocampus in both hemispheres and both age-groups, compared with the respective age-matched control values. In contrast, ischemia-hyperoxia only caused decreases in the level of Glu and increases in Lac in both age-groups, with inappreciable changes in the other metabolites (Tables 5, 6). These alterations appeared grossly similar in both age-groups.

## Discussion

In the present study, measurements of a series of cerebral metabolites were performed by ^1^H-MRS to establish the tissue alterations following hypoxic or hyperoxic exposure performed on the background of ischemic insult. The major finding of the study is that cerebral ischemia associated with hypoxia caused derangement of the energy-related content of brain metabolites which was conspicuously more pronounced in the hippocampal than cortical areas. In the hippocampus, ischemia associated with hypoxia reduced the content of all brain metabolites studied, and the effects were more evident in the aged animals. Alterations in brain metabolites were unrelated to the ischemia-injured hemisphere, as they were about equally distributed in the respective areas of both hemispheres. The ischemia-hyperoxia procedure caused much less dedicated changes in the brain metabolites, which may reflect more limited tissue damage.

It is known that oxidative stress is a relevant mechanism involved in the process of brain aging [4,5]. Moreover, aging is the most important risk factor for neurodegenerative disorders, such as Alzheimer and Parkinson's disease [16,17]. Oxidative damage is essential for most neurodegenerative diseases [2,3,16,18]. Excessive production of ROS also is germane to the neuronal damage associated with ischemia and brain edema, ranging from metabolic alterations to apoptosis or necrosis [8,19]. Hypoxia and hyperoxia, the former being often a sequel of a disease process and the latter a treatment modality, act as inducers of ROS formation [6-8]. In the present study, therefore, we set out to investigate the age-differences in the content of brain metabolites in response to varying oxygen supply on the background of ischemic insult. To this end we developed a model of cerebral ischemia associated with exposure to chronic hypoxia and hyperoxia in two contrasting age-groups of rats, young and senescent, in which selected metabolites were quantified by means of proton magnetic resonance spectroscopy.

The ultimate goal of the present study was the identification of biochemical markers of oxidative stress in the brain. That goal was not really achieved, as alterations in metabolites were overall modest and variably different. However, some basic pattern of metabolic brain alterations was brought out. The results demonstrate no significant age-dependent regional differences in the brain content of metabolites, between young and aged rats in the normoxic condition, except for the higher levels of Lac and mI in the hippocampus of the aged rats. These findings are consistent with the data reported in other *ex vivo *studies [12,13]. Nevertheless, the increase in Lac is an interesting finding in that the Lac level in the hippocampus might be considered a useful marker of aging. Indeed, increased Lac levels have been found in the brains of healthy elderly people, as measured by ^1^H-MRS [20]. Also, high levels of Lac have been found in the brains of humans affected by pre-senile dementia [21] or Alzheimer's disease [22].

The present finding of reduced Lac concentration in the hippocampus of ischemia-hypoxia-treated animals, particularly evident in the aged animals, is in agreement with our previously reported data [12]. The finding is, however, at variance with the data reported by Higuchi et al [23] who found increased Lac concentrations following global ischemia. However, one important point to consider is the duration of the ischemia-hypoxic procedure, which in our study lasted for 24 h. It is probable that during this longer period lactic acid, locally produced by neurons, was cleared by the circulatory system and that the neuronal death due to combination of hypoxia with ischemia ultimately resulted in a reduction of Lac formation. On the other hand, increased hippocampal Lac concentration following ischemia-hyperoxia may reflect a milder insult resulting only in neuronal damage rather than death induced by hypoxia.

That hippocampus is a brain region particularly susceptible to metabolic derangement is confirmed by a marked decrease in NAA concentration following the ischemia-hypoxia insult. Reductions in NAA levels have been found after neuronal damage or dysfunction, even in the absence of neuronal death [24]. Moreover, an age-related decrease in NAA has been found in human cortical gray matter [25,26]. It is conceivable that ischemia-hyperoxia is a milder disturbance than ischemia-hypoxia, inasmuch as the former did not cause any changes in the hippocampal NAA concentration, at least as revealed by the ^1^H-MRS resolution, which is a well known index of neural viability. In the hippocampus, only modest reductions of Glu levels were found after ischemia-hyperoxia, whereas its substantial decreases occurred bilaterally following ischemia-hypoxia. These findings are consistent with the hypothesis that ischemia-hypoxia causes a marked release of endogenous Glu, thereby reducing its tissue content measured by ^1^H-MRS. However, it is impossible to establish to what extent the reduced Glu concentration induced by ischemia-hypoxia might be an index of neuronal damage. In addition, all other metabolites measured (i.e., GABA, mI, choline, and Cre) were found to be significantly reduced in the hippocampus of both young and aged rats after the ischemia-hypoxia procedure, whereas ischemia associated with hyperoxia did not cause any significant changes in the content of these metabolites.

Unlike the hippocampal area that, according to our findings, was much more susceptible to the effects of hypoxic than hyperoxic treatment after antecedent cerebral ischemia, the fronto-parietal and occipital cortices were similarly sensitive to both treatments. However, metabolic alterations in both cortical areas were modest, compared with those in the hippocampus. Lac concentrations increased in both cortical areas by both treatments, but the effects of hypoxia were stronger than those of hyperoxia, in both young and aged rats. This difference probably reflects the prevalence of anaerobic metabolism when ischemia is associated with hypoxia, whereas hyperoxia may compensate, in part, for the deleterious effects of ischemia. The increase in Lac in cortical areas reflects neuronal damage, since unchanged or slightly modified levels of NAA and Glu rather rule out the occurrence of neuronal death. A similar trend was followed by GABA, whose levels were reduced in the fronto-parietal cortex. Depletion of Glu and GABA tissue concentrations induced by hypoxia in the fronto-parietal cortex are probably consequent to their release elicited by the ischemic insult [27,28]. In contrast, GABA concentration was significantly increased in the occipital cortex of both young and aged rats after ischemia-hyperoxia.

There is a spate of pathological conditions in which cerebral ischemia may ensue; most notably strokes or thromboembolic brain events, transient ischemic attacks, or neurodegenerative disorders. Both hypoxia and hyperoxia are frequent accompaniments of cerebral ischemia in clinical settings. Therefore, the study of the effects on brain metabolites of a combination of either gas condition with ischemia seemed warranted. Hypoxia may be antecedent to brain event, such as in chronic hypoxic lung pathologies, exemplified by obstructive pulmonary disease or sleep-related breathing disorders which, in fact, sharply increase the risk for brain ischemic events [29], or may develop as a sequel of breathing disorders secondary to brain ischemia. Either way, hypoxia appears a major detriment to brain energy metabolites as shown in the present study. Hyperoxia, on the other hand, is often used as a pharmacological tool to alleviate ischemic symptoms.

There are a number of limitations to this study. Histological and functional correlates of the cerebral ischemia induced were not traced, nor was the brain tissue redox status assessed. The study also was thought out as basically non-invasive during the 24-h period of the delivery of inspired gas mixtures; therefore, no arterial blood gas content and acid-base status were controlled. Furthermore, spectroscopic measurements were carried out in brain tissue *ex vivo *and the extrapolation of the results to *in vivo *conditions is not fully applicable. The resolution of these issues would require alternative study designs.

## Conclusions

Despite the limitations and although the exact determinants of metabolic alterations in the brain are unsettled, we believe we have shown that the association of hypoxia and cerebral ischemia impairs brain metabolism and may be a particular detriment for the hippocampus-controlled functions; for instance, memory and emotions [30]. As hyperoxia associated with ischemia appears to have no major brain tissue damaging effects, the study does not disapprove a judicial use of O_2_-enriched inspiratory gas mixtures to alleviate symptoms accompanying cerebral ischemia. A better understanding of the mechanisms underlying metabolic brain changes associated with hypoxia and hyperoxia during ischemic insults is essential to facilitate recognition of the optimum health-related strategies for ischemia.

## Competing interests

The authors declare that they have no competing interests.

## Authors' contributions

MAM conceived of the study, and participated in its design and coordination. NDA carried out the proton magnetic resonance spectroscopy. CDG carried out the hypoxic and hyperoxic exposures. PDI carried out the ischemia-hypoxia and ischemia-hyperoxia experimental conditions. SDL participated in the study and performed the statistical analysis. PG participated in the sample preparation and the extraction of metabolites. EE participated in conception and design of the study. MP performed the analysis and interpretation of data and was involved in writing the manuscript and revising it critically for scientific content. All authors read and approved the final manuscript.
